# The Effect of Pneumococcal, Influenza, and COVID-19 Vaccinations on COVID-19 Hospitalization and Progression in People over 65 Years Old Living in Nursing Homes

**DOI:** 10.3390/vaccines11050943

**Published:** 2023-05-04

**Authors:** Feyza Kutay Yilmaz, Mustafa Cakir, Hatice Ikiisik, Isil Maral

**Affiliations:** Department of Public Health, Faculty of Medicine, Istanbul Medeniyet University, Istanbul 34700, Turkey; mustafa.cakir@medeniyet.edu.tr (M.C.); hatice.ikiisik@medeniyet.edu.tr (H.I.); isil.maral@medeniyet.edu.tr (I.M.)

**Keywords:** COVID-19, COVID-19 vaccine, influenza vaccine, pneumococcal vaccine, nursing home

## Abstract

Infectious diseases pose a major threat to elderly populations. Streptococcus pneumonia bacteria, influenza-causing viruses, and COVID-19 viruses cause three pathologies in the respiratory system with similar symptoms, transmission routes, and risk factors. Our study aimed to evaluate the effects of pneumococcal, influenza, and COVID-19 vaccinations on the status of COVID-19 hospitalization and progression in people over 65 years of age living in nursing homes. This study was performed in all nursing homes and elderly care centers in the Uskudar district of Istanbul.The diagnosis rate of COVID-19 was determined as 49%, the rate of hospitalization as 22.4%, the rate of hospitalization in the intensive care unit as 12.2%. The rate of intubation was determined as 10.4%, the rate of mechanical ventilation as 11.1% and the rate of COVID-19 related mortality rate as 9.7%. When the factors affecting the diagnosis of COVID-19 were examined, the presence and dose of COVID-19 vaccine was protective. When the factors affecting hospitalization status were examined, male sex and presence of chronic disease were risk factors; four doses of COVID-19 vaccine and influenza vaccine and pneumococcal vaccine together with COVID-19 vaccine were protective. When the factors affecting COVID-19-related death were examined, the male sex was a risk factor; the pneumococcal and influenza vaccine together with COVID-19 vaccine were protective. Our results revealed that the availability of influenza and pneumococcal vaccines positively impacted the progression of COVID-19 disease in the elderly population living in nursing homes.

## 1. Introduction

Infectious diseases pose a major threat to elderly populations. Functional and structural changes in tissues and organs with age, immune system suppression, accompanying systemic diseases cause the elderly to become more vulnerable to infections. Moreover, increasing hospitalizations and increasing resistant pathogens due to additional diseases, difficulties in diagnosis and inability to start treatment on time cause severe respiratory tract infections in the elderly [1]. Streptococcus pneumonia bacteria, influenza-causing viruses, and COVID-19 viruses cause three pathologies in the respiratory system with similar symptoms, transmission routes, and risk factors. The simultaneous coexistence of these pathogens in people over 65 years of age may worsen the existing progression, and the immunosuppression of any of these infections may increase susceptibility to other infections [2,3,4,5]. Furthermore, the body’s fight against these infections in people over the age of 65 may increase addiction and the need for care and reduce the quality of life [6].

One factor affecting older people’s vulnerability to infectious diseases is the use of health and care facilities, which are communal living spaces. For infectious diseases, communal areas such as schools, dormitories, elderly care and rehabilitation centers, military units, prisons, and immigrant camps are areas where the risk of exposure and transmission is higher, and those living in these areas are at higher risk than the rest of society [7,8,9]. In addition, those staying in long-term care facilities are a vulnerable and disadvantaged group, both medically and socially. They can be severely affected by these diseases due to their age and susceptibility to comorbid diseases [7]. For all these reasons, considering that COVID-19 vaccine supply may be limited at the beginning, the WHO has requested that the vaccination of disadvantaged groups at greater risk be given priority [10].

Immunization is the most effective way to prevent severe outcomes of these infections. Due to the ever-changing nature of influenza viruses, the WHO recommends that especially at-risk groups (children between 6 months and 5 years old, individuals over 65 years of age, those with chronic diseases, pregnant women, and healthcare workers) be vaccinated annually [11,12,13]. In its recommendations published in 2021, the ACIP mentioned that the 2021–2022 flu season coincided with the COVID-19 pandemic, and that preventing seasonal flu or reducing its severity through vaccination will alleviate hospital admissions, hospitalizations, and, indirectly, the burden of the COVID-19 pandemic on the health system [3]. In the 2022 recommendations, it is stated that the severity of the next flu season cannot be predicted. Therefore, vaccination will be crucial for controlling severe respiratory diseases during the ongoing COVID-19 pandemic [14].

Polysaccharide pneumococcal vaccine (PPSV) and conjugated pneumococcal vaccine (PCV) have been developed to protect against invasive and noninvasive diseases caused by pneumococci, especially pneumonia. The CDC recommends that people over the age of 65 get the pneumococcal vaccine. In Turkey, PCV13 is administered free of charge in family health centers (FHCs) [1,15]. Since pneumococcal infections cause high morbidity and mortality in the elderly, individuals over 65 years of age are included in the target population [16].

People over 65 are at risk for all three pathologies. For this reason, influenza, pneumococcal, and COVID-19 vaccines are recommended and administered free of charge by the Ministry of Health in Turkey. Reducing the risk of severe progression of these diseases, which have serious morbidity and mortality in the elderly and vulnerable populations, will protect the elderly individuals in the vulnerable group. It will also alleviate the burden on the health system and provide economical use of medical resources [3,11,12,16].

This study aimed to evaluate the effects of pneumococcal, influenza, and COVID-19 vaccinations on contracting COVID-19 infection, hospitalization, and disease progression in people over 65 years of age living in nursing homes. 

## 2. Materials and Methods

### 2.1. Study Design and Participants

This study was performed in 9 nursing homes and elderly care centers in the Uskudar district of Istanbul. It was planned as a retrospective cohort. The data in the first part of the survey were collected through face-to-face interviews between 01 December 2021 and 31 January 2022. The data in the second part of the survey were obtained from health information systems between 01 December 2021 and 31 March 2022. The period investigated in the study is between 11 March 2020, when the first case of COVID-19 was observed in Turkey, and 31 March 2022, the deadline determined by the researchers to collect data. To accurately examine the effect of immunization on the diagnosis and progression of COVID-19 in the population studied and to minimize possible sources of error, people who lived in nursing homes between the examined dates but died before the face-to-face survey were also included in the study. The information for these people was obtained through health information systems. Health information systems were also used to confirm the accuracy of data collected face-to-face to eliminate recall bias.

In the study, data were collected with two methods. The first of these was the data obtained through a face-to-face survey. The researcher administered a face-to-face questionnaire to the residents of 9 nursing homes and elderly care centers in the Uskudar district who consented to the study. People who did not volunteer to participate in the study, who were under the age of 65, whose mental status was not sufficient, and whose health condition was poor enough to receive palliative care were excluded, and 450 people were reached for the face-to-face survey method (total 547 people; 82.5%). The second data acquisition method in the study was data obtained from health information records. In this section, COVID-19 diagnosis, follow-up information, and immunization information were included. COVID-19 diagnosis, follow-up and immunization information of 450 people was obtained through health information systems. By including the information of 187 people (only data obtained through health information systems) who died in nursing homes for any reason during the period when the study was carried out, the information of 637 people in total was acquired. 

### 2.2. Survey Items

In the first part of the survey, conducted face to face, information about age, sex, education level, the number of people sharing the same room, the place where s/he ate the most, frequency of use of social space, and chronic disease information were questioned. In the second part of the survey (through health information systems), COVID-19 diagnosis and follow-up information (COVID-19 diagnosis status/time, presence of symptoms, hospitalization status/time/duration of hospital stay, intensive care admission/time/duration of intensive care stay, intubation status/time) was questioned. RT-PCR test positivity in the system was based on for the diagnosis of COVID-19. In addition, in the second part, immunization status [presence/time/type/dose of pneumococcal/influenza/COVID-19 vaccine (BioNTech/SinoVac)] was investigated. The information in the first part of the survey was also double-checked from the health system records. Influenza vaccine information of 52 people who died in 2020 (including the first months of 2021) due to systemic deficiencies could not be accessed and was not included in the study. This is indicated by the “total number (n)” in the sections related to influenza vaccine in the findings section. 

### 2.3. Statistical Analysis

The SPSS 22.0 program was used for statistical analysis. Descriptive statistics were presented as percentage, mean, and standard deviation values for continuous and normally distributed data, and median and 25–75 percentile values for non-normally distributed data. The chi-square test was used for the comparison of categorical data and the Student-*t* test compared numerical variables. The predictive effect of significant variables in univariate analyzes of the diagnosis of COVID-19 infection and hospitalization was evaluated with Cox regression analysis. Cox regression analysis was also used to evaluate the COVID-19-related mortality differentials. *p* < 0.05 was considered statistically significant.

## 3. Results

It was determined that 58.9% were women, 21% were in the age range of 65–74, 33.3% were in the age range of 75–84, and 45.7% were 85 years old and over. The mean age was 82.4 ± 8.9 years (median: 84, min–max: 65–101), and 92% had at least one chronic disease. The most common chronic diseases were hypertension at a rate of 74.7%, cardiovascular disease at 39.9%, and hyperlipidemia at 33.6%. The sociodemographic characteristics of the individuals and other information about their social lives are presented in Table 1.

Among the 637 people included in the study, the frequency of diagnosis of COVID-19 was 49%, hospitalization was 22.4%, admission to the intensive care unit was 12.2%, intubation was 10.4%, mechanical ventilation was 11.1%, and the COVID-19-related mortality rate was 9.7% (Table 2). 

It was determined that 24.4% of 312 people diagnosed in Turkey were diagnosed in 2020 as of March, when the first COVID-19 case was announced; 61.5% were diagnosed in 2021, and 14.1% had been diagnosed through March 2022 (including March). Of the 312 diagnosed patients, 45.8% were hospitalized. Furthermore, 54.5% of the 143 patients who were hospitalized were admitted to the intensive care unit, 46.2% were intubated, 49.7% needed mechanical ventilation, and ECMO was applied to only one person (Table 2).

**Figure 1:**
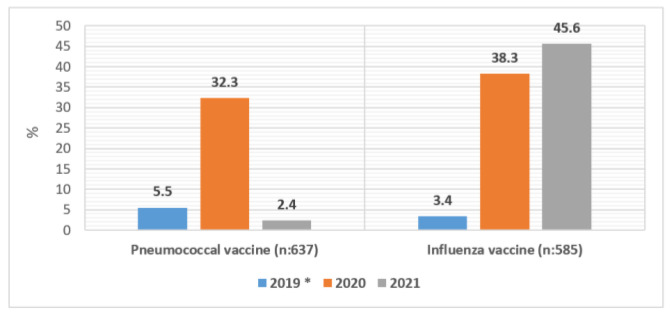
Vaccination status by years (%), over 65, nursing home, Uskudar-Istanbul, March 2022. *: Denotes year 2019 and before for pneumococcal vaccine.

It was determined that 40.2% of the participants had the pneumococcal vaccine. The frequency of those vaccinated in 2019 and before was 5.5%, and the frequency of those vaccinated in 2020 was 32.3%. It was determined that 63.7% of people had at least one dose of influenza vaccine. The frequency of vaccination was 38.3% and 45.6% in 2020 and 2021, respectively (Figure 1). 

While 85.7% of the 637 people included in the study had at least one dose of the COVID-19 vaccine, 91 people had never received a COVID-19 vaccine. There were people who had four doses of the COVID-19 vaccine, with a maximum of 46.6%. When the three vaccines were evaluated together, some people had the COVID-19+influenza+pneumococcal vaccine at most at 29.4%, then the COVID-19+influenza vaccine at 27.2% and only the COVID-19 vaccine with 21.9%. 23 people were not vaccinated against all three diseases.

It was determined that 29.4% (*n*: 187) of the 637 people whose information was accessed consisted of people who stayed in the specified nursing homes and died for any reason. It was determined that 51.3% of the deceased were women. The mean age was 84.7 ± 8.5 years, being the median as 86 (min: 65–max: 101). It was determined that 90.9% of them had at least one chronic disease. It was stated that the cause of death of 33.2% (*n*: 62) was related to COVID-19, and the cause of death of 66.8% (*n*: 125) was a natural death.

It was observed that men had more pneumococcal vaccines than women, and those aged 65–74 years compared to 85 years and over. In addition, it was determined that secondary school graduates had more pneumococcal vaccines than other education levels, and those who stayed with three or more people compared to those who stayed alone (*p*: <0.001, *p*: 0.017, *p*: 0.046, *p*: 0.006, respectively). Besides, 51.7% of those vaccinated against influenza and 43.2% of those vaccinated against COVID-19 also received the pneumococcal vaccine (*p* < 0.001) (Table 3). 

It was determined that secondary school graduates had more influenza vaccines than other education levels, and those who stayed in a double room compared to those who stayed alone (*p*: 0.014, *p*: 0.005, respectively). Moreover, 78.5% of those vaccinated against pneumococcus and 66.2% of those vaccinated against COVID-19 had influenza vaccination (*p* < 0.001) (Table 3).

It was observed that women were vaccinated with COVID-19 vaccine more than man. It was determined that the 65–74 and 75–84 age group had more COVID-19 vaccines than 85 years and over. The frequency of COVID-19 vaccination at all education levels was over 97% (*p*: 0.049, *p*: 0.009, *p*: 0.524, respectively). Moreover, 92.2% of those vaccinated against pneumococcus and 96.5% vaccinated against influenza were also vaccinated against COVID-19 (*p* < 0.001). 187 people had all three vaccines, and 23 people did not have all three vaccines (Table 3).

**Figure 2:**
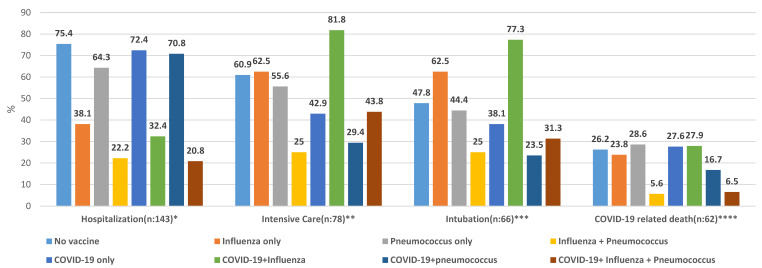
Distribution of COVID-19 progression stages of patients diagnosed with COVID-19 by vaccination status at time of diagnosis (%), over 65, nursing home, Uskudar-Istanbul, March 2022. *: Difference in hospitalization is between those who have never been vaccinated and those who have influenza + pneumococcal vaccine; those who have COVID-19 + influenza vaccine and those who have only COVID-19 vaccine; those who have received COVID-19 + influenza + pneumococcal vaccine and those who have received COVID-19 + pneumococcal vaccine (*p*: <0.001). **: The difference for intensive care is between COVID-19 + influenza, COVID-19 + pneumococcal, and COVID-19 + influenza + pneumococcal vaccines (*p*: 0.034). ***: The difference for intubation is between COVID-19 + influenza, COVID-19 + pneumococcal, and COVID-19 + influenza + pneumococcal vaccines (*p*: 0.028). ****: The difference in COVID-19-related death is between those who have never been vaccinated, those who have had COVID-19 + influenza and COVID-19 + influenza + pneumococcal vaccines (*p*: 0.015).

The distribution of COVID-19 diagnosis and progression stages according to sociodemographic characteristics and current vaccination status is presented in Table 4 and Figure 2. It was observed that among the people with a diagnosis of COVID-19 who were hospitalized, men, those over 85 years old, and those with chronic diseases had more intensive care admissions (*p*: 0.054, *p*: 0.748, *p*: 0.330, respectively). It was determined that those who got the pneumococcal vaccine together with the COVID-19 vaccine were admitted to the intensive care unit less than those who received the influenza vaccine together with the COVID-19 vaccine and those who received all three vaccines (*p*: 0.034) (Table 4, Figure 2). Similarly, men, those over 85 years of age, and those with chronic diseases were more frequently intubated (*p*: 0.206, *p*: 0.733, *p*: 0.124, respectively). It was determined that those who got the pneumococcal vaccine together with the COVID-19 vaccine were intubated less than those who received the influenza vaccine together with the COVID-19 vaccine and those who received all three vaccines (*p*: 0.028) (Table 4, Figure 2).

When the factors affecting the diagnosis status of COVID-19 were evaluated; male sex, being a secondary school graduate, staying in double rooms, using social areas all the time, and having only pneumococcal vaccine at the time of diagnosis was determined to be risky. Having a university or higher education degree, eating in the cafeteria, presence of COVID-19 vaccine available at the time of diagnosis was protective (Model 1–2). Moreover, increasing the dose of the COVID-19 vaccine increased the protection (Model 1), and influenza and/or pneumococcal vaccines available together with the COVID-19 vaccine were also observed to be protective against diagnosis (Model 2).

When the factors affecting the hospitalization status were evaluated; it was determined that the male sex and the presence of chronic disease were risky (Model 1–2). Moreover, the influenza vaccine available at the time of diagnosis, and four doses of COVID-19 vaccine were protective (Model 1). In addition, the protective effect of the influenza vaccine, together with the COVID-19 vaccine at the time of diagnosis, increased with the addition of the pneumococcal vaccine (Model 2).

When the factors affecting the COVID-19-related death situation were examined; it was determined that the male sex was risky (Model 1–2). In addition, the pneumococcal vaccine available at the time of diagnosis was protective (Model 1). Besides, the availability of pneumococcal and influenza vaccines, together with the COVID-19 vaccine, was also protective (Model 2).

In Table 5 and Table 6, Cox was not included as a variable in the regression because all of the COVID-19-related deaths had a chronic disease.

## 4. Discussion and Conclusions

The present study examined the effects of COVID-19, influenza, and pneumococcal vaccines on the status and progression stages of COVID-19 disease in people over 65 living in nursing homes. To examine this effect, people over the age of 65 in all nursing homes and elderly care centers in the Uskudar district were selected as the population. 

In our study, 40.2% of the participants had received pneumococcal vaccine, 63.7% had at least one dose of flu vaccine, 85.7% had at least one dose of COVID-19 vaccine. While the frequency of pneumococcal vaccines in 2019 and before was 5.5%, the frequency of those who were vaccinated in 2020 was 32.3% and increased. The frequency of influenza vaccination was 38.3% and 45.6% in 2020 and 2021, respectively. Studies by Breuker et al., Zhou et al., and Loubet et al., have shown that individuals with chronic diseases and at risk for pneumococcal, influenza, and COVID-19 diseases have increased awareness of communicable diseases with pandemics and epidemics. The tendency of these individuals to be vaccinated against pneumococcus and influenza have increased compared to the past [17,18,19]. According to the meta-analysis in which 27 studies were included, the frequency of influenza vaccination increased with the pandemic worldwide, regardless of factors such as region, age, and sex [20]. Parallel to the studies, there was an increase in the frequency of those who had pneumococcal and influenza vaccination in 2020 and 2021 in our study. This result may be due to the increased interest and awareness of vaccine-preventable diseases, immunization, and the orientation towards health-seeking behaviors during pandemics. The COVID-19 pandemic may have positively affected self-responsibility regarding health, and awareness of protection may have increased. 

The frequency of obtaining influenza and pneumococcal vaccines was lower in many studies compared to our study [4,21,22,23,24]. These studies included not only the nursing home population but also all elderly individuals in the community. Elderly care centers where elderly individuals live collectively can encourage the behavior of obtaining other adult vaccines, especially the COVID-19 vaccine. In our study, the frequency of the pneumococcal vaccine was lower than the influenza vaccine. Sharing and repeating information tools about the flu vaccine with the public in October-November every year may have increased awareness.

In our study, pneumococcal and influenza vaccination status was higher those aged 65–74 years. This group, which is younger than other age groups, may be more prone to access health-related information and may be more conscious of it. In addition, the increase in the number of people staying together has positively affected pneumococcal and influenza vaccination behavior. In the study by Zhang et al., elderly people living with others were more willing to be vaccinated [25]. Increased social contact facilitates communication and increases the possibility of obtaining health-related information. 

The frequency of getting the COVID-19 vaccine was 97% and above, regardless of educational status and social life behaviors. Being in an active pandemic may explain the high incidence of COVID-19 vaccination. Staying in nursing homes, especially for the COVID-19 vaccine, may have provided an advantage and affected the frequency of vaccination, as they are in an institution that guides people’s health-related decisions. İn addition, our study revealed that vaccination and non-vaccination behavior could affect each other. In the study of Ikiısık et al., it was mentioned that it is an expected result that COVID-19 vaccine acceptance, whose transmission pattern and clinical features are similar, is higher in those who receive the regular flu vaccine every year [26]. 

A systematic review examining 80 articles conducted in 2020 stated that less-crowded nursing homes are less risky in terms of transmission [27]. Similarly, a systematic review examining 36 studies conducted in 2020–2021 found that large facilities have a higher probability of cases than small facilities [28]. Our study concluded that the number of people staying in a room and the frequency of using social areas affect the diagnosis of COVID-19. Nursing homes surveyed in our study applied certain rules to nursing home residents, staff, and visitors to minimize contact and comply with physical distancing rules during the COVID-19 period. These measures taken in nursing homes, which host a vulnerable group that will be affected earliest and most severely by a pandemic, were established in parallel with measures implemented and recommended in many countries [29]. 

In our study, having a university or higher education was also observed to be protective against a diagnosis of COVID-19. High education level may have affected protective practices such as masks, physical distancing, and attention to hygiene measures and may have reduced the incidence of COVID-19 disease. However, being a secondary school graduate was a risk factor in the diagnosis of COVID-19. In addition to the effect of education level on health behaviors, the fact that secondary school graduates mostly stay in rooms with two or three people may have affected the result.

According to the results of our research, the COVID-19 vaccine is protective against diagnosis, and the increase in the dose of the COVID-19 vaccine has increased its protection. The presence of only the COVID-19 vaccine revealed the greatest protection, and the pneumococcal and influenza vaccines together with the COVID-19 vaccine were also protective of a diagnosis. According to the interim analysis results for Sinovac (CoronaVac) and the completed phase 3 study results of the mRNA vaccine Pfizer-BioNTech, fewer COVID-19 cases were detected in the vaccine group compared to the placebo group [30,31]. Our results were consistent for these two vaccines, the efficacy and safety of which were supported by phase 3 studies and applied in Turkey.

Some studies have reported that older adults vaccinated with the pneumococcal vaccine have a lower risk of COVID-19 infection [32,33]. Our study concluded that the pneumococcal vaccine increased the risk of being diagnosed. The effect of the number of people staying together on the transmission of infection may have overshadowed the effect of the vaccine. Besides, communal living areas such as nursing homes are places where it is difficult to maintain social distance and control the frequency of contact. The number of contacts, duration, intensity, and type of contact are criteria that increase the possibility of transmission [34].

Candelli et al. concluded that the influenza vaccine has a reducing effect on catching COVID-19 infection [22]. The fact that nursing homes were chosen in our study may have created a more favorable environment for the spread of infection compared to the group over 65 years old in Candelli’s study. Nursing homes are also environments where people often come together with various social activities for their physical and psychological health. This situation may be risky for the transmission of COVID-19. A meta-analysis including a total of 16 studies demonstrated that being vaccinated for influenza is associated with a lower risk of COVID-19 infection [35]. The tendency to pay more attention to the health behaviors of those who have received the flu vaccine may have also resulted in better compliance with COVID-19 prevention measures. Our study observed that the influenza vaccine had a protective effect on diagnosis when present together with the COVID-19 vaccine, although not alone.

When the factors affecting hospitalization were examined, the influenza vaccination and four doses of the COVID-19 vaccine were protective. The protective effect of the influenza vaccine together with the COVID-19 vaccine at the time of diagnosis increased with the addition of the pneumococcal vaccine. 

In the study of Griffin et al., which included people over the age of 16, it was observed that unvaccinated individuals were hospitalized at a higher rate than partially and fully vaccinated individuals with the COVID-19 vaccine [36]. Although only individuals over the age of 65 were included in our study, and our population consisted of individuals at higher risk of hospitalization due to age and comorbid diseases, our results are consistent. In addition, some studies involving the elderly population have similarly shown that COVID-19 vaccines reduce hospitalizations [37,38].

In the study of Lewnard et al., it was concluded that the pneumococcal vaccine might have a protective effect on the diagnosis of COVID-19, hospitalization, and mortality [39]. In our study, positive results of the pneumococcal vaccine were observed in hospitalization, intensive care, intubation, and COVID-19-related death. The fact that pneumococci and SARS-CoV-2 affect the same systems with similar symptoms may have enabled the vaccine to slow the worsening progression. In addition, the transmission of these diseases occurs in similar ways. Chowdhury et al. indicated a regression in invasive pneumococcal disease during the pandemic and attributed this to the public health measures implemented due to COVID-19 [4]. Therefore, it is crucial to keep the pneumococcal vaccine coverage high to reduce the possible effects of the periods when public health measures are relaxed during the COVID-19 pandemic [4]. Also, COVID-19 and pneumococcal diseases are caused by two respiratory pathogens that are affected by similar risk factors, such as age and comorbid disease. Being infected with COVID-19 is also a risk factor for pneumococcal disease, as it weakens immune function [5]. Therefore, it is vital to increase the immunization behaviors of risky groups during the COVID-19 pandemic.

The study by Ragni et al., the study of Conlon et al., it was stated that people with a diagnosis of COVID-19 who received the influenza vaccine were less likely to be hospitalized [23,24]. In a meta-analysis study in which 23 articles were examined, the risk of hospitalization was lower in those who had an influenza vaccine [40]. Although our results were similar to the literature, our study covered not only the period when COVID-19 vaccines were not developed, but also both periods. Over time, new variant types emerged. In addition, our study was conducted with a population over 65 years of age and with high comorbidities. All these factors made it difficult to talk about the direct effect of the influenza vaccine. 

In the study of Fink et al., the need for intensive care treatment was 7% lower, and the mortality rate was 16% lower in patients recently vaccinated for influenza compared to those not vaccinated [41]. Our study determined no relationship between the influenza vaccine administered in the last year and hospitalization in the intensive care unit and death. There is a temporal difference between the study in Brazil and our study. The most of the temporal interval included in our study coincides with the time when COVID-19 vaccines are available. Furthermore, the age range included in the study in Brazil is wider. In our study, the age range was narrower, and the population was a group with comorbid diseases. Therefore, our population may be riskier regarding intensive care and death compared to the study of Fink et al.

The studies of Stańczak-Mrozek et al. and Marín-Hernández et al. revealed that the influenza vaccine reduces the risk of death in COVID-19 patients over 65 years of age [42,43]. According to a meta-analysis study evaluating the results of 15 studies, the outcome was similar [44]. In our study, the co-existed of 3 vaccines revealed protection in terms of COVID-19-related death. This result may show that influenza and pneumococcal vaccines available together with the COVID-19 vaccine may have a possible benefit on progression, although there is no direct effect of the influenza vaccine.

One of the problems encountered during the ongoing pandemic is that both COVID-19 and influenza show a wide range of disease course and the disease burden is high. It is possible that seasonal flu will exacerbate the effects of the COVID-19 pandemic. It is also possible that COVID-19 may reinvigorate and exacerbate seasonal flu [45]. To control the burden on countries caused by the simultaneous COVID-19 and flu pandemics called the ‘Twindemic’, access to vaccines for these diseases should be supported by public health and social measures [46,47,48].

Despite all recommendations and public health policies worldwide, immunization for both influenza and pneumococcus is below the desired level in most countries and should be increased. The immunization behavior acquired during the pandemic can be turned into an advantage to increase the coverage of these vaccines [47]. According to the 2018 report by the ECDC, the 75% vaccination target could not be achieved by remaining below 50% in chronically ill and elderly individuals, while according to the report by the CDC evaluating data from the 2020–2021 season, the vaccination coverage for those over 65 years of age has increased and approached optimal [49,50].

Although the direct associations between COVID-19 and influenza/pneumococcal vaccines are minimal, revealing the potential benefits during the pandemic period may encourage annual vaccination of people over 65 years of age. To increase vaccine coverage, it is crucial to increase and facilitate access to vaccines. The idea of co-administration of vaccines may provide benefits such as simplifying the immunization program, reducing costs, access advantages, and patient compliance [47]. Currently, the co-administration of COVID-19 and influenza vaccine is in question in the vaccination programs of many countries. WHO also supports this idea because of its potential benefits [51]. This method may become more effective by reminding the pneumococcal vaccines when the vaccines are administered together. Strategies for these purposes should be organized and awareness should be raised in the community about the benefits and necessity of influenza and pneumococcal vaccines.

Although nursing homes may be advantageous in providing other vaccines, especially the COVID-19 vaccine, higher vaccination levels should be targeted for all three vaccines. Consideration should also be given to the immunization status of staff or caregivers in close contact with vulnerable. Nursing homes should be seen as an advantage for transmitting the correct information on immunization. Training should be planned to increase the health literacy of elderly individuals. Considering that individual experiences can positively affect the decision to be vaccinated, peer education can be applied. It can be conducted in the form of transferring the experiences of people who have had severe influenza, invasive pneumonia, or severe COVID-19 to elderly individuals. Besides, data on vaccines should be systematically collected, access to the target audience should be ensured, reminder systems should be developed, scope gaps should be determined.

Our study was conducted in nursing homes in a single district in one province. This is a limitation in that our results reflect that population. Multicenter studies may yield more effective results. Our study was conducted only with people over the age of 65 staying in nursing homes. This is another limitation in that our results reflect the population over 65 years of age. Future research including the general population may contribute to the literature. Similar studies in the literature generally belong to the year 2020, when the COVID-19 vaccine was not yet widely applied. These studies evaluated the efficacy of only one of the influenza or pneumococcal vaccines. Our study is strong in terms of evaluating all three vaccines together.

## Figures and Tables

**Table 1:** Distribution of Sociodemographic Characteristics and Social Life Information, Over 65, Nursing Home, Uskudar-Istanbul, March 2022.

		*n*	%
Sex (*n*: 637 *)	Female	375	58.9
	Male	262	41.1
Age group (*n*: 637)	65–74	134	21.0
	75–84	212	33.3
	85 and over	291	45.7
Chronic disease status (*n*: 637)	Yes	586	92.0
	No	51	8.0
Educational status (*n*: 450 **)	Illiterate	9	2.0
	Primary school graduate	112	24.9
	Middle school graduate	73	16.2
	High school graduate	169	37.6
	University/Masters/PhD	87	19.3
Number of people in the room in the nursing home (*n*: 450)	1	214	47.6
	2	168	37.3
	3 and above	68	15.1
Place to eat (*n*: 450)	In own room	157	34.9
	In the cafeteria	293	65.1
Frequency of using social areas (*n*: 450)	None	91	20.2
	Rarely (1–2 times a week)	65	14.4
	Sometimes (3–5 times a week)	50	11.1
	Often (at least once most days of the week)	53	11.8
	Always (At least once a day)	191	42.4

*: The deceased are also included. **: Only the information for people staying in nursing homes at the time of data collection is available.

**Table 2:** Distribution of COVID-19 Diagnoses by Patient Follow-up Information in the Diagnosis Period, Over 65, Nursing Home, Uskudar-Istanbul, March 2022.

		*n*	%
Status of COVID-19 diagnosis * (*n*: 637 *)	Yes	312	49.0
	No	325	51.0
COVID-19 diagnosis time (*n*: 312)	Year 2020 (from March 2020)	76	24.4
	Year 2021	192	61.5
	Year 2022 [Until March 2022 (including March)]	44	14.1
Patient follow-up status during the diagnosis process (*n*: 312)	Hospitalized (with symptoms)	143	45.8
	Isolation in nursing home/outpatient (no symptoms/scan)	169	54.2
Admission to the intensive care unit during the diagnosis process (*n*: 143)	Yes	78	54.5
	No	65	45.5
Intubation status during the diagnosis process (*n*: 143)	Yes	66	46.2
	No	77	53.8
Need for mechanical ventilation (*n*: 143)	Yes	71	49.7
	No	72	50.3
Information on COVID-19 progression (*n*: 637 *)	COVID-19 diagnosis	312	49.0
	Hospitalization	143	22.4
	Intensive care	78	12.2
	Intubation	66	10.4
	Mechanical ventilation	71	11.1
	COVID-19-related death **	62	9.7

*: Deceased persons are also included. **: All of the COVID-19-related deaths were hospitalized before intensive care.

**Table 3:** Distribution of Pneumococcal, Influenza, and COVID-19 Vaccines by Sociodemographic Characteristics, Social Life Information, and Other Vaccines, Over 65, Nursing Home, Uskudar-Istanbul, March 2022.

		Pneumococcal Vaccine (*n*: 637 *)			Influenza Vaccine (*n*: 585 **)			COVID-19 Vaccine (*n*: 637 *)		
		Yes	No	*p*	Yes	No	*p*	Yes	No	*p*
		*n* (%)	*n* (%)		*n* (%)	*n* (%)		*n* (%)	*n* (%)	
Sex (*n*: 637 *)	Female	126 (33.6)	249 (66.4)	<0.001	218 (62.6)	130 (37.4)	0.496	330 (88.0)	45 (12.0)	0.049
	Male	130 (49.6)	132 (50.4)		155 (65.4)	82 (34.6)		216 (82.4)	46 (17.6)	
Age group (*n*: 637)	65–74	67 (50.0) ^a^	67 (50.0)	0.017	85 (66.9)	42 (33.1)	0.600	121 (90.3) ^a^	13 (9.7) ^a^	0.009
	75–84	86 (40.6)	126 (59.4)		128 (64.3)	71 (35.7)		189 (89.2) ^a^	23(10.8) ^a^	
	85 and over	103 (35.4) ^b^	188 (64.6)		160 (61.8)	99 (38.2)		236 (81.1) ^b^	55 (18.9) ^b^	
Chronic disease status (*n*: 637)	Yes	233 (39.8)	353 (60.2)	0.456	347 (64.0)	195 (36.0)	0.640	502 (85.7)	84 (14.3)	0.905
	No	23 (45.1)	28 (54.9)		26 (60.5)	17 (39.5)		44 (86.3)	7 (13.7)	
Educational status (*n*: 450)	Illiterate/primary school	45 (37.2)	76 (62.8)	0.046	91 (75.2)	30 (24.8)	0.014	120 (99.2)	1 (0.8)	0.524
	Middle school graduate	41 (56.2)	32 (43.8)		59 (80.8)	14 (19.2)		72 (98.6)	1 (1.4)	
	High school and above	66 (39.1)	103 (60.9)		109 (64.5)	60 (35.5)		164 (97.0)	5 (3.0)	
	Uni./MA/PG	39 (44.8)	48 (55.2)		54 (62.1)	33 (37.9)		86 (98.9)	1 (1.1)	
Number of people staying together (*n*: 450)	1 (alone)	77 (36.0) ^a^	137 (64.0)	0.006	134 (62.6) ^a^	80 (37.4)	0.005	211 (98.6)	3 (1.4)	0.705
	2	75 (44.6)	93 (55.4)		131 (78.0) ^b^	37 (22.0)		165 (98.2)	3 (1.8)	
	3 and above	39 (57.4) ^b^	29 (42.6)		48 (70.6)	20 (29.4)		66 (97.1)	2 (2.9)	
Place to eat (*n*: 450)	Own room	71 (45.2)	86 (54.8)	0.383	106 (67.5)	51 (32.5)	0.491	155 (98.7)	2 (1.3)	0.719
	Cafeteria	120 (41.0)	173 (59.0)		207 (70.6)	86 (29.4)		287 (98.0)	6 (2.0)	
Frequency of using social areas (*n*: 450)	None/rare	67 (42.9)	89 (57.1)	0.265	103 (66.0)	53 (34.0)	0.327	154 (98.7)	2 (1.3)	0.842
	Sometimes/often	50 (48.5)	53 (51.5)		77 (74.8)	26 (25.2)		101 (98.1)	2 (1.9)	
	Always	74 (38.7)	117 (61.3)		133 (69.6)	58 (3.04)		187 (97.9)	4 (2.1)	
Pneumococcal vaccine (*n*: 637)	Yes				193 (78.5)	53 (21.5)	<0.001	236 (92.2)	20 (7.8)	<0.001
	No				180 (53.1)	159 (46.9)		310 (81.4)	71 (18.6)	
Influenza vaccine (*n*: 585)	Yes	193 (51.7)	180 (48.3)	<0.001				360 (96.5)	13 (3.5)	<0.001
	No	53 (25.0)	159 (75.0)					184 (86.8)	28 (13.2)	
COVID-19 vaccine (*n*: 637)	Yes	236 (43.2)	310 (56.8)	<0.001	360 (66.2)	184 (33.8)	<0.001			
	No	20 (22.0)	71 (78.0)		13 (31.7)	28 (68.3)				

*: Deceased persons are also included. **: Excludes those for whom influenza vaccine information was not available. %: The column percentages are given in Table 3. Different letters associated with post hoc results represent the difference between groups.

**Table 4:** Distribution of COVID-19 Diagnosis and Progression Stages by Sociodemographic Characteristics and Existing Vaccine Status, Over 65, Nursing Home, Uskudar-Istanbul, March 2022.

			COVID-19 Diagnosis (*n*: 312)			Hospitalization (*n*: 143)			Intensive Care (*n*: 78)			Intubation (*n*: 66)			COVID-19-Related Death (*n*: 62)		
			*n* (%)	*n* (%)	*p*	*n* (%)	*n* (%)	*p*	*n* (%)	*n* (%)	*p*	*n* (%)	*n* (%)	*p*	*n* (%)	*n* (%)	*p*
Sex		Female	177 (47.2)	198 (52.8)	0.282	71 (40.1)	106 (59.9)	0.020	33 (46.5)	38 (53.5)	0.054	29 (40.8)	42 (59.2)	0.206	28 (15.8)	149 (84.2)	0.040
		Male	135 (51.5)	127 (48.5)		72 (53.3)	63 (46.7)		45 (62.5)	27 (37.5)		37 (51.4)	35 (48.6)		34 (25.2)	101 (74.8)	
Age group		65–74	70 (52.2)	64 (47.8)	0.686	29 (41.4)	41 (58.6)	0.249	14 (48.3)	15 (51.7)	0.748	13 (44.8)	16 (55.2)	0.733	12 (17.1)	58 (82.9)	0.457
		75–84	103 (48.6)	109 (51.4)		43 (41.7)	60 (58.3)		24 (55.8)	19 (44.2)		18 (41.9)	25 (58.1)		18 (17.5)	85 (82.5)	
		85+	139 (47.8)	152 (52.2)		71 (51.1)	68 (48.9)		40 (56.3)	31 (43.7)		35 (49.3)	36 (50.7)		32 (23.0)	107 (77.0)	
Chronic disease status		Yes	294 (50.2)	292 (49.8)	0.042	139 (47.3)	155 (52.7)	0.038	77 (55.4)	62 (44.6)	0.330	66 (47.5)	73 (52.5)	0.124	62 (21.1)	232 (78.9)	0.029
		No	18 (35.3)	33 (64.7)		4 (22.2)	14 (77.8)		1 (25.0)	3 (75.0)		0 (0)	4 (100)		0 (0)	18 (100)	
All Available Vaccines	Pneumoc. vaccine	Yes	133 (53.8)	114 (46.2)	0.051	46 (34.6)	87(65.4)	0.001	18 (39.1)	28 (60.9)	0.011	14 (30.4)	32 (69.6)	0.009	14 (10.5)	119 (89.5)	<0.001
		No	179 (45.9)	211 (54.1)		97 (54.2)	82 (45.8)		60 (61.9)	37 (38.1)		52 (53.6)	45 (46.4)		48 (26.8)	131 (73.2)	
	Influenza vaccine *	Yes	184 (52.9)	164 (47.1)	0.257	50(27.2)	134 (72.8)	<0.001	31 (62.0)	19 (38.0)	0.060	28 (56.0)	22 (44.0)	0.014	30 (16.3)	154 (83.7)	0.508
		No	114 (48.1)	123 (51.9)		82 (71.9)	32 (28.1)		37 (45.1)	45 (54.9)		28 (34.1)	54 (65.9)		22 (19.3)	92 (80.7)	
	COVID-19 Vaccine	Yes	198 (41.6)	278 (58.4)	<0.001	76 (38.4)	122 (61.6)	0.001	39 (51.3)	37 (48.7)	0.409	34 (44.7)	42 (55.3)	0.717	36 (18.2)	162 (81.8)	0.324
		No	114 (70.8)	47 (29.2)		67 (58.8)	47 (41.2)		39 (58.2)	28 (41.8)		32 (47.8)	35 (52.2)		26 (22.8)	88 (77.2)	

Information about the deceased persons is also included in the table. * Excludes those for whom influenza vaccine information was not available. %: Column percentages are given in Table 4.

**Table 5:** Factors Affecting COVID-19 Diagnosis, Hospitalization, and COVID-19-Related Deaths, Over 65, Nursing Home, Uskudar-Istanbul, March 2022.

MODEL 1			COVID-19 Diagnosis (*n*: 213 *)		COVID-19 Diagnosis (*n*: 312)		Hospitalization (*n*: 143)		COVID-19-Related Death (*n*: 62)	
			HR (95% CI)	*p*	HR (95% CI)	*p*	HR (95% CI)	*p*	HR (95% CI)	*p*
Sex		Female	Ref	0.020	Ref	0.004	Ref	0.014	Ref	0.002
		Male	1.444 (1.06–1.96)		1.422(1.11–1.81)		1.570 (1.09–2.25)		2.381 (1.38–4.08)	
Age group		65–74	Ref	0.911	Ref	0.924	Ref	0.231	Ref	0.637
		75–84	0.972 (0.67–1.39)	0.880	1.027 (0.75–1.40)	0.868	0.990 (0.61–1.60)	0.969	1.028 (0.49-2.15)	0.942
		85+	1.044 (0.72-1.49)	0.817	1.062 (0.78-1.44)	0.700	1.347 (0.85-2.12)	0.200	1.303 (0.65-2.58)	0.448
Chronic disease status		Yes	1.085 (0.59-1.97)	0.789	1.038 (0.62-1.73)	0.887	3.511 (1.28-9.61)	0.015	-	-
		No	Ref		Ref		Ref		-	
Educational status		Illiterate/primary school	Ref	0.000	-	-	-	-	-	-
		Secondary school gr.	1.545 (1.04–2.28)	0.029	-		-		-	
		High school and above	0.715 (0.50–1.02)	0.065	-		-		-	
		Uni./MA/PG	0.581 (0.36–0.92)	0.023	-		-		-	
Number of people staying together		1 (alone)	Ref	0.004	-	-	-	-	-	-
		2	1.389 (1.01–1.90)	0.041	-		-		-	
		3 and above	0.733 (0.46–1.14)	0.175	-		-		-	
Place to eat		Own room	Ref	0.002	-	-	-	-	-	-
		Cafeteria	0.556 (0.38–0.80)		-		-		-	
Frequency of using social areas		None/rare	Ref	0.010	-	-	-	-	-	-
		Sometimes/often	0.944 (0.61–1.44)	0.789	-		-		-	
		Always	1.629 (1.08–2.45)	0.019	-		-		-	
All Available VaccinesAvailable Vaccines	Pneumococcal vaccine	Yes	1.686 (1.23–2.30)	0.001	1.319 (1.02–1.69)	0.032	0.799 (0.54–1.16)	0.242	0.362 (0.19–0.68)	0.002
		No	Ref		Ref		Ref		Ref	
	Influenza vaccine	Yes	0.875 (0.64–1.19)	0.403	1.188 (0.92–1.52)	0.179	0.291 (0.19–0.43)	0.000	0.745 (0.40–1.36)	0.341
		No	Ref		Ref		Ref		Ref	
	COVID-19 vaccine	Yes	0.046 (0.03–0.06)	0.000	-	-	-	-	-	-
		No	Ref		-		-		-	
	COVID-19 vaccine dose	0	-	-	Ref	0.000	Ref	0.027	Ref	0.183
		1	-		0.320 (0.18–0.54)	0.000	1.810 (0.97–3.36)	0.060	1.171 (0.34–3.99)	0.801
		2	-		0.306 (0.23–0.40)	0.000	1.014 (0.65–1.56)	0.949	1.662 (0.86–3.18)	0.125
		3	-		0.041 (0.02–0.06)	0.000	0.674 (0.34–1.32)	0.252	0.894 (0.30–2.60)	0.837
		4	-		0.016 (0.01–0.02)	0.000	0.393 (0.16–0.93)	0.035	0.207 (0.02–1.57)	0.129
		5	-		0.000 (0.00–0.02)	0.977	-		-	

HR: Hazard ratio. *: Deceased persons are not included.

**Table 6:** Factors Affecting COVID-19 Diagnosis, Hospitalization, and COVID-19-Related Deaths, Over 65, Nursing Home, Uskudar-Istanbul, March 2022.

MODEL 2		COVID-19 Diagnosis (*n*: 213 *)		COVID-19 Diagnosis (*n*: 312)		Hospitalization (*n*: 143)		COVID-19-Related Death (*n*: 62)	
		HR (95% CI)	*p*	HR (95% CI)	*p*	HR (95% CI)	*p*	HR (95% CI)	*p*
Sex	Female	Ref	0.038	Ref	0.548	Ref	0.014	Ref	0.002
	Male	1.388 (1.01–1.89)		1.078 (0.84–1.37)		1.576 (1.09–2.26)		2.385 (1.36–4.16)	
Age group	65–74	Ref	0.895	Ref	0.316	Ref	0.144	Ref	0.355
	75–84	1.070 (0.73–1.56)	0.725	0.904 (0.66–1.23)	0.529	0.937 (0.57–1.51)	0.791	0.981 (0.46–2.06)	0.959
	85+	1.091 (0.75–1.57)	0.643	1.111 (0.82–1.50)	0.492	1.358 (0.86–2.14)	0.187	1.449 (0.72–2.89)	0.293
Chronic disease status	Yes	1.088 (0.59–1.98)	0.785	1.067 (0.64–1.77)	0.803	3.292 (1.20–9.00)	0.020	-	-
	No	Ref		Ref		Ref		-	
Educational status	Illiterate/primary school	Ref	0.000	-	-	-	-	-	-
	Secondary school gr.	1.505 (1.01–2.23)	0.043	-		-		-	
	High school and above	0.697 (0.48–1.00)	0.051	-		-		-	
	Uni./MA/PG	0.569 (0.35–0.92)	0.022	-		-		-	
Number of people staying together	1 (alone)	Ref	0.010	-	-	-	-	-	-
	2	1.437 (1.04–1.97)	0.026	-		-		-	
	3 and above	0.824 (0.52–1.30)	0.409	-		-		-	
Place to eat	Own room	Ref	0.000	-	-	-	-	-	-
	Cafeteria	0.480 (0.32–0.70)		-		-		-	
Frequency of using social areas	None/rare	Ref	0.005	-	-	-	-	-	-
	Sometimes/often	1.014 (0.65–1.56)	0.951	-		-		-	
	Always	1.777 (1.217–2.69)	0.007	-		-		-	
All Available Vaccines	No vaccine	Ref	0.000	Ref	0.000	Ref	0.000	Ref	0.025
	Influenza only	0.441 (0.23–0.84)	0.103	0.894 (0.53–1.49)	0.666	0.340 (0.15–0.72)	0.005	1.078 (0.38–3.00)	0.885
	Pneumococcus only	3.349 (1.10–10.19)	0.033	3.016 (1.47–6.15)	0.002	0.729 (0.35–1.52)	0.399	1.007 (0.32–3.07)	0.991
	Influenza + pneumococcus	0.578 (0.29–1.13)	0.110	0.761 (0.44–1.31)	0.328	0.218 (0.07–0.61)	0.004	0.261 (0.03–2.00)	0.196
	COVID-19 vaccine only **	0.016 (0.00–0.03)	0.000	0.045 (0.02–0.07)	0.000	0.830 (0.49–1.40)	0.485	1.231 (0.52–2.89)	0.635
	COVID-19 + influenza	0.033 (0.01–0.05)	0.000	0.097 (0.06–0.14)	0.000	0.290 (0.17–0.49)	0.000	1.455 (0.71–2.94)	0.297
	COVID-19 + pneumococcus	0.057 (0.02–0.11)	0.000	0.122 (0.07–0.19)	0.000	0.965 (0.54–1.69)	0.902	0.708 (0.23–2.13)	0.540
	COVID-19 + influenza + pneumococcus	0.046 (0.02–0.07)	0.000	0.102 (0.07–0.14)	0.000	0.166 (0.09–0.29)	0.000	0.222 (0.08–0.61)	0.003

HR: Hazard ratio. *: Deceased persons are not included. **: Indicates the presence of at least 1 dose of the COVID-19 vaccine.

## Data Availability

Not applicable.
